# Sirtuin signalling governs calcium homeostasis and mitochondrial function in metabolic syndrome cardiomyocytes

**DOI:** 10.1113/EP094062

**Published:** 2026-08-03

**Authors:** Firat Akat, Leila Aryan, Suatnur Şık, Erkan Tuncay

**Affiliations:** ^1^ Department of Physiology, Faculty of Medicine Ankara University Ankara Turkiye; ^2^ Department of Biophysics, Faculty of Medicine Ankara University Ankara Turkiye; ^3^ Stem Cell Institute Ankara University Ankara Turkiye; ^4^ Institute of Health Sciences Ankara University Ankara Turkiye; ^5^ Department of Interdisciplinary Neuroscience, Faculty of Medicine Ankara Turkiye

**Keywords:** calcium, calcium homeostasis, cardiomyocytes, metabolic syndrome, mitochondrial dysfunction, oxidative stress, sarcoplasmic reticulum, SIRT1, sirtuin

## Abstract

Metabolic syndrome (MetS) is a major contributor to cardiovascular disease and is characterized by impaired Ca^2+^ handling and mitochondrial dysfunction in cardiomyocytes. However, the upstream mechanisms linking metabolic stress to these alterations remain incompletely defined. Here, we investigated whether sirtuin signalling coordinates intracellular Ca^2+^ homeostasis and mitochondrial function in a high‐sucrose diet‐induced mouse model of MetS. Male BALB/c mice were exposed to 32% sucrose for 24 weeks, followed by isolation of ventricular cardiomyocytes. MetS cardiomyocytes exhibited mitochondrial depolarization, increased reactive oxygen species production, elevated basal cytosolic Ca^2+^ levels, reduced Ca^2+^ transient amplitude and decreased sarcoplasmic reticulum Ca^2+^ content. These alterations were associated with reduced phospholamban phosphorylation, increased CaMKII and ryanodine receptor phosphorylation and activation of pro‐apoptotic signalling. Notably, pharmacological inhibition of SIRT1 with EX527 in control cardiomyocytes recapitulated key features of the MetS phenotype, whereas SIRT1 activation with SRT1720 restored mitochondrial membrane potential, reduced reactive oxygen species production, improved Ca^2+^ handling, normalized aberrant phosphorylation of Ca^2+^‐handling proteins and attenuated apoptotic signalling. Collectively, these findings identify SIRT1 as a critical integrator of mitochondrial function and Ca^2+^ homeostasis during metabolic stress, highlighting sirtuin‐dependent pathways as promising therapeutic targets in metabolic cardiomyopathy.

## Abstract (highlights)

- **What is the central question of this study?**
  Does impaired SIRT1 signalling contribute to mitochondrial dysfunction and disrupted intracellular Ca^2+^ homeostasis in cardiomyocytes during metabolic syndrome?
- **What is the main finding and its importance?**
  SIRT1 inhibition in control cardiomyocytes reproduced key features of the metabolic syndrome phenotype, whereas SIRT1 activation improved mitochondrial function, oxidative stress, Ca^2+^ handling and apoptotic signalling in MetS cardiomyocytes. These findings identify SIRT1 as a mechanistic link between metabolic stress, mitochondrial dysfunction and impaired Ca^2+^ homeostasis, supporting its potential as a therapeutic target in metabolic cardiomyopathy.

## 1 INTRODUCTION

Metabolic syndrome (MetS) refers to a cluster of metabolic risk factors that markedly increase the risk of cardiovascular disease. These risk factors include abdominal obesity, hypertriglyceridaemia, reduced HDL cholesterol, high blood pressure and elevated fasting blood glucose (Alberti et al., 2009; Durak et al., 2022; Tuncay, Olgar et al., 2019). Although there is an ongoing debate regarding its diagnostic criteria, it is generally accepted that the presence of at least three of these risk factors is required for a diagnosis of MetS (Nolan et al., 2017; Tune et al., 2017). Given that MetS substantially increases the risk of developing type 2 diabetes mellitus, understanding the mechanisms linking metabolic alterations to cardiovascular dysfunction is of major importance for public health. MetS represents a major global health burden, affecting ∼20%–25% of the adult population worldwide, with prevalence varying according to region and diagnostic criteria (Ranasinghe et al., 2017). The strong association between MetS and adverse cardiovascular outcomes highlights its importance as a key focus of cardiovascular research. As a result, therapeutic strategies primarily target metabolic disturbances or aim to protect the heart from MetS‐induced injury (Mottillo et al., 2010; Tune et al., 2017).

Cardiac contractile function critically depends on intracellular calcium homeostasis (Bers, 2002, 2008). In MetS, alterations in Ca^2+^‐handling proteins and their post‐translational modifications disrupt Ca^2+^ homeostasis, impair contractile function and increase arrhythmogenic risk (Kuo et al., 2025). For instance, ryanodine receptor 2 (RyR2) dysregulation disrupts excitation–contraction coupling, resulting in reduced Ca^2+^ transients and impaired contractility, whereas decreased SERCA2a function impairs sarcoplasmic reticulum Ca^2+^ reuptake and diastolic relaxation (Louch et al., 2012). Oxidative stress and mitochondrial dysfunction have been increasingly implicated in impaired Ca^2+^ handling in MetS, in part through disrupted mitochondria–Ca^2+^ crosstalk (Sommese et al., 2016).

Sirtuins (SIRTs) are NAD^+^‐dependent class III deacetylases that act as metabolic sensors by regulatingthe acetylation of histone and non‐histone proteins in response to cellular energy status (Imai & Guarente, 2014). In cardiomyocytes, sirtuins have emerged as important modulators of mitochondrial function and intracellular Ca^2+^ homeostasis. Altered sirtuin activity has been reported in insulin resistance and MetS, leading to dysregulated protein acetylation and impaired cardiac function (Schug & Li, 2011). Notably, acetylation of Ca^2+^‐handling proteins such as sarcoplasmic reticulum Ca^2+^‐ATPase (SERCA2a) directly modulates Ca^2+^ cycling and contractile performance, whereas restoration of SIRT1 activity improves SERCA2a function and Ca^2+^ homeostasis in diabetic cardiomyopathy models (Sulaiman et al., 2010).

Given the emerging role of sirtuin signalling as an integrative regulator of metabolic stress responses, the aim of the present study was to determine whether altered SIRT1 activity contributes to dysregulated Ca^2+^ handling and mitochondrial dysfunction in a high‐sucrose diet‐induced model of MetS. We hypothesized that MetS‐induced mitochondrial dysfunction and disruption of intracellular Ca^2+^ homeostasis are mediated, at least in part, by altered SIRT1 activity and can therefore be modulated through pharmacological inhibition or activation of SIRT1.

## 2 MATERIALS AND METHODS

### 2.1 Ethical approval

Animals used in this study were procured from the Ankara University Experimental Animals Breeding and Research Laboratory. All experimental procedures were reviewed and approved by the Ankara University Animal Experiments Local Ethics Committee (approval number: 2023‐16‐155). All procedures adhered to the *Guide for the Care and Use of Laboratory Animals* (Albus, 2012) and the ARRIVE Guidelines 2.0 (Percie du Sert et al., 2020). Appropriate anaesthesia and humane euthanasia procedures were applied, and every effort was made to minimize animal suffering and distress throughout the study.

Male, adult (10‐week‐old) BALB/C mice were maintained at a constant temperature (22°C –24°C) and humidity (50%–55%) and with a 12 h light–12 h dark cycle throughout the experiments. Animals had ad libitum access to standard rodent chow and tap water. After a 1 week adaptation period, animals were randomly assigned into two groups: control (Con) and MetS.

### 2.2 Induction of MetS

The initial body weight and blood glucose levels of the animals did not differ between the groups. Animals in the MetS group received a sucrose solution (32% w/v) instead of tap water for 24 weeks. Commercially available food‐grade sucrose was used. The sucrose concentration and exposure period were selected based on our previous studies (Durak et al., 2018; Okatan et al., 2015, 2016). The sucrose solution for these animals was refreshed at least twice per week, and the drinking bottles were washed thoroughly to remove any residual sugar and prevent bacterial contamination. Total fluid and food intake of animals in all groups were monitored throughout the study.

We evaluated the metabolic state of the animals using several parameters, including body weight, blood glucose level, glucose tolerance, liver function and dyslipidaemia.

To assess glucose tolerance, we performed an intraperitoneal glucose tolerance test (IPGTT). For the IPGTT, animals were fasted for 6–8 h. After the fasting period, a 50% (w/v) glucose solution was administered intraperitoneally at a dose of 2 g/kg. Blood glucose levels were measured before injection and at specific time points (15, 30, 60 and 120 min) using a glucometer (On Call Plus Glucometer; Acon Labs Inc., USA) after making a small incision at the tip of the tail. A glucose–time curve was then plotted, and the area under the curve was compared between the groups.

Serum ALT (Alanine aminotransferase), AST (Aspartate transaminase), total cholesterol, LDL (Low‐density lipoprotein), HDL (High‐density lipoprotein) and triglyceride levels were measured using a biochemistry autoanalyzer (Fujifilm Dri‐Chem NX600) as indicators of liver function and dyslipidaemia.

### 2.3 Termination of the experiments and cardiomyocyte isolation

At week 24 of the experimental protocol, animals were anaesthetized by intraperitoneal administration of ketamine (90 mg/kg) combined with xylazine (10 mg/kg). Adequate general anaesthesia was confirmed by the loss of the pedal withdrawal reflex. Animals were then killed by exsanguination under deep general anaesthesia. The hearts were rapidly excised, rinsed, and immediately immersed in ice‐cold Ca^2+^‐free isolation solution containing (mM): NaCl, 117; KCl, 5.7; NaHCO_3_, 4.4; KH_2_PO_4_, 1.5; MgCl_2_, 3.6; HEPES, 20; glucose, 11.7; and taurine, 20. Excess connective tissue was carefully removed, heart weights were recorded, and the tissue was processed without delay for cardiomyocyte isolation.

Following heart excision, blood samples were collected and centrifuged at 5000 x *g* for 5 min at 4°C. The resulting serum was aliquoted and stored at −80°C until subsequent biochemical analyses.

Ventricular cardiomyocytes were isolated using a Langendorff perfusion‐based enzymatic dissociation technique, as previously described, with minor modifications (Tuncay et al., 2023). Briefly, hearts were cannulated via the ascending aorta and mounted on a Langendorff perfusion system. All perfusion solutions were maintained at 37°C and continuously oxygenated with 100% O_2_ throughout the procedure. The hearts were initially perfused with Ca^2+^‐free isolation solution to remove residual blood, followed by perfusion with a collagenase solution containing collagenase type II (1 mg/mL; Worthington Biochemicals) prepared in Ca^2+^‐free isolation solution. Enzymatic digestion was terminated by perfusion with Ca^2+^‐free isolation solution supplemented with bovine serum albumin (1 mg/mL) to remove residual collagenase activity. After completion of enzymatic perfusion, the hearts were removed from the Langendorff apparatus, and the ventricular tissue was carefully dissected and gently triturated in Ca^2+^‐free isolation solution. The resulting cell suspension was filtered through a nylon mesh to eliminate undigested tissue fragments and incubated at 37°C. Cardiomyocytes were then gradually adapted to physiological calcium concentrations by repeated resuspension at 5 min intervals in solutions containing incrementally increasing Ca^2+^ levels, allowing calcium tolerance and preservation of cell viability.

Isolated cardiomyocytes obtained from control and MetS groups were subsequently subjected to pharmacological modulation of sirtuin activity. Cardiomyocytes isolated from control animals were maintained either without treatment or exposed to the selective SIRT1 inhibitor EX527 (Sigma Aldrich; E7034) at a concentration of 10 µM for a duration of 3 h, whereas cardiomyocytes obtained from MetS animals were maintained either without treatment or treated with the SIRT1 activator SRT1720 (Sigma‐Aldrich; 567860) at a concentration of 2 µM for 3 h. Following these incubation procedures, cardiomyocytes were collected and used for the assessment of mitochondrial membrane potential (MMP), reactive oxygen species (ROS) production, intracellular Ca^2+^ handling and protein expression analysis by western blotting.

### 2.4 Measurement of MMP

The MMP was assessed using a fluorescence‐based approach, as previously described (Billur et al., 2016). Isolated ventricular cardiomyocytes were incubated with the cationic, membrane‐permeant dye JC‐1 (5 µM, 30 min; at 37°C; Invitrogen, T3168), which exhibits potential‐dependent aggregation in polarized mitochondria. Fluorescence imaging was performed using a confocal microscope (Leica TCS SP5) with excitation at 488 nm. Emission signals corresponding to the monomeric (535 ± 15 nm) and J‐aggregate (585 ± 15 nm) forms of JC‐1 were acquired to calculate the red/green fluorescence ratio as an index of MMP. Full depolarization was induced by applying the uncoupling agent carbonyl cyanide 4‐(trifluoromethoxy) phenylhydrazone (FCCP; 5 µM; Sigma‐Aldrich; C2920) for calibration.

### 2.5 Measurement of ROS

ROS levels in cardiomyocytes were assessed using the fluorescent probe 2′,7′‐dichlorodihydrofluorescein diacetate (DCFDA; 10 µM, Invitrogen, D399) and imaged with a confocal microscope (Leica TCS SP5). Excitation and emission wavelengths of 488 and 530 ± 15 nm, respectively, were used for ROS detection. Basal fluorescence was recorded, after which cells were exposed to 100 µM H_2_O_2_ to elicit maximal ROS formation and enable indirect quantification. ROS levels were calculated by comparing the increase in fluorescence between basal measurements and the calibrated donor‐induced responses (Olgar et al., 2020; Tuncay, Bitirim et al., 2019).

### 2.6 [Ca^2+^]_i_ and Ca^2+^ transient measurements

Intracellular transient calcium changes were assessed in isolated cardiomyocytes loaded with the fluorescent Ca^2+^ indicator Fura‐2 AM (Fura‐2AM Cell Permeant Sigma Aldrich 344905; 3 µM), as previously described (Turan et al., 1996). Measurements were performed at room temperature (21°C ± 2°C). Fluorescence signals were acquired using a microspectrofluorometer equipped with FELIX software (Photon Technology International, Inc., NJ, USA). Cells were stimulated via electrical field stimulation (10 ms pulses at 0.2 Hz), and Fura‐2 fluorescence was recorded at dual‐excitation wavelengths of 340/380 nm, with emission collected at 510 nm. The *F*_340_/ *F*_380_ fluorescence ratio was calculated, and the amplitude of the Ca^2+^ transient (peak minus basal ratio) was used as an index of intracellular free Ca^2+^ dynamics for each cell. To assess total sarcoplasmic reticulum Ca^2+^ content, 10 mM caffeine (Sigma Aldrich; 27600) was applied rapidly, and the resulting Ca^2+^ release was recorded as the caffeine‐evoked Ca^2+^ response.

### 2.7 Western (immuno‐)blot analysis

After the incubation step, cells were lysed using NP‐40 lysis buffer (250 mM NaCl, 1% NP‐40 and 50 mM Tris–HCl, pH 8.0, supplemented with 1 × Protease inhibitor cocktail). The supernatants obtained after centrifugation (12 000*g*, 5 min, 4°C) were collected, and protein concentrations were determined using the BCA assay kit (Thermo Scientific Pierce, Waltham, MA, USA). Equal amounts of protein were separated on SDS–polyacrylamide gels, transferred onto polyvinylidene fluoride membranes, blocked with bovine serum albumin, and incubated with primary antibodies against SERCA2 (Cell Signaling, #9580), Ca^2+^/calmodulin‐dependent protein kinase II (CaMKII) (Cell Signaling, #4436), phospho‐CaMKII (Thr286) (Cell Signaling, #12716), ryanodine receptor (Badrilla, #A010_35AP), phospho‐ryanodine receptor (Ser2808) (Badrilla, #A010_31AP), phospholamban (Cell Signaling, #14562), phospho‐phospholamban (Ser16/Thr17) (Cell Signaling, #8496), BCL2 (ThermoFisher, #pa5‐27094), Bax (Biolegend, #633602), Beclin (Biolegend, #O93F3) and β‐actin (Cell Signaling, #4970), following the manufacturers’ protocols. Specific bands were visualized using suitable horseradish peroxidase‐conjugated secondary antibodies and detected with the ImmunoCruz Western Blotting Luminol Reagent (Santa Cruz, sc‐2048). Band intensities were quantified using ImageJ software (Tuncay et al., 2017; Tuncay, Bitirim 2019).

### 2.8 Data analysis and statistics

All datasets were initially assessed for normality using the Shapiro–Wilk test. Variables that met parametric assumptions were analysed using appropriate parametric tests. For comparisons involving more than two groups, one‐way ANOVA was performed, followed by Tukey's *post hoc* test when significant main effects were detected. For western blot analyses and other datasets that did not meet parametric assumptions, the Kruskal–Wallis test followed by Dunn's multiple‐comparison *post hoc* test were used. For comparisons at single time points between Con and MetS groups, Student's unpaired *t*‐test was used. Repeatedly measured parameters, such as blood glucose levels during the IPGTT, were analysed using repeated‐measures ANOVA. All statistical analyses were conducted using GraphPad Prism (v.8.1; GraphPad Software, San Diego, CA, USA). Data are expressed as the mean ± SD, and statistical significance was set at *P* < 0.05.

## 3 RESULTS

### 3.1 Sucrose feeding induced marked metabolic disturbances

**FIGURE 1:**
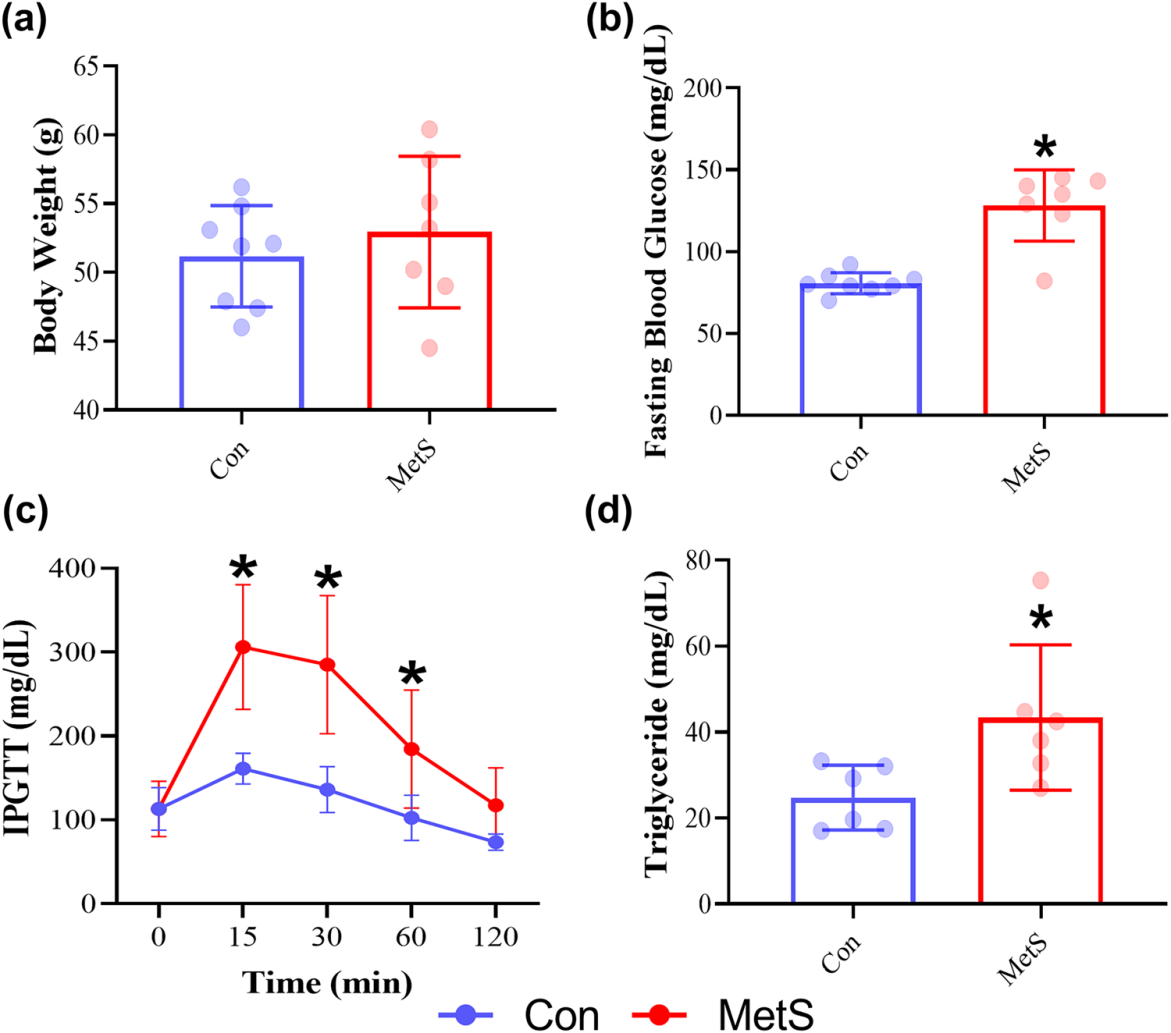
Metabolic characterization of experimental animals. (a) Terminal body weight. (b) Terminal fasting blood glucose level. (c) IPGTT. (d) Serum triglyceride level. Data are presented as the mean ± SD. Individual data points represent individual animals. Sample sizes were as follows: Con (*n* = 8) and MetS (*n* = 7). *P < 0.05 vs. Con. Abbreviations: Con, control; IPGTT, intraperitoneal glucose tolerance test; MetS, metabolic syndrome.

We assessed several metabolic parameters to verify the development of MetS. Terminal body weight, fasting blood glucose levels, intraperitoneal glucose tolerance and serum triglyceride concentrations are summarized in Figure 1.

Body weight did not differ significantly among the experimental groups (*P* = 0.4729). In contrast, fasting blood glucose levels were significantly elevated in the MetS group compared with control animals (*P* < 0.0001). Moreover, MetS animals exhibited a pronounced impairment in glucose tolerance, reflecting reduced insulin responsiveness. During the IPGTT, blood glucose levels were significantly higher in the MetS group than in controls, particularly at 15, 30 and 60 min after glucose administration. Two‐way repeated‐measures ANOVA revealed significant effects of time [*F*(4,52) = 42.18, *P *< 0.0001], group [*F*(1,13) = 23.52, *P* = 0.0003] and time × group interaction [*F*(4,52) = 11.99, *P *< 0.0001]. Consistent with these findings, the area under the glucose curve was markedly increased in the MetS group compared with controls (23 678 vs. 13 148 mg.min/dl). Finally, serum triglyceride levels were markedly increased in MetS animals compared with the Con group (*P* = 0.0334). Collectively, these findings confirm that sucrose feeding induced a robust metabolic dysfunction consistent with the development of MetS.

Other MetS‐related parameters, including ALT, AST, HDL, LDL and total cholesterol levels, did not differ between Con and MetS animals (data not shown).

### 3.2 Metabolic stress impairs mitochondrial function and redox balance

**FIGURE 2:**
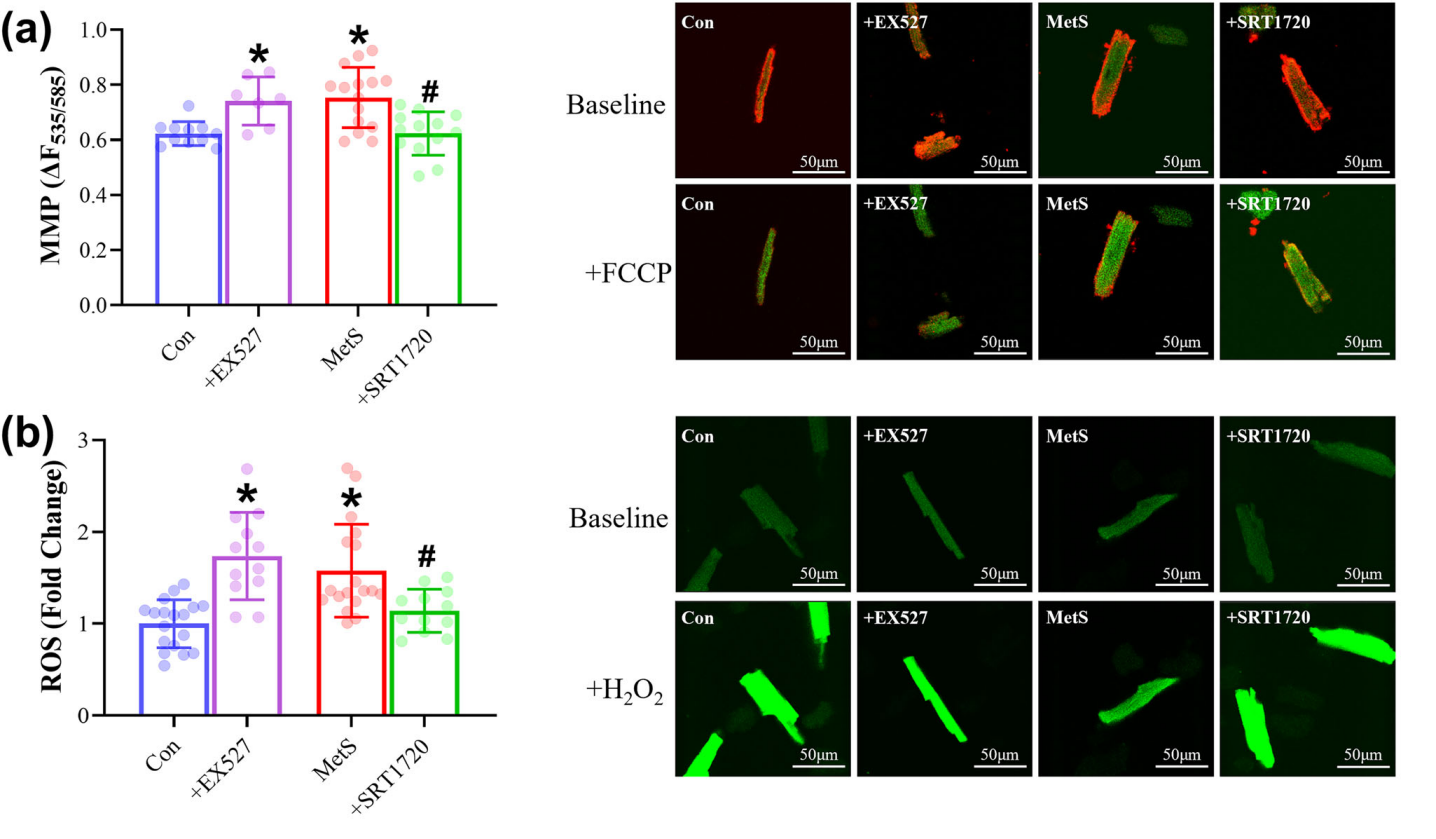
MMP and ROS production in experimental groups. (a) MMP. (b) ROS levels. Data are presented as the mean ± SD. Individual data points represent individual cells obtained from separate cell isolations. Sample sizes were as follows: MMP, Con (*n* = 11 cells), EX527 (*n* = 7 cells), MetS (*n* = 15 cells) and SRT1720 (*n* = 13 cells); ROS, Con (*n* = 17 cells), EX527 (*n* = 12 cells), MetS (*n* = 18 cells) and SRT1720 (*n* = 12 cells). *P < 0.05 vs. Con; #*P* < 0.05 vs. MetS. Abbreviations: Con, control; MetS, metabolic syndrome; MMP, mitochondrial membrane potential; ROS, reactive oxygen species.

MMP and intracellular ROS levels are presented in Figure 2.

Analysis of MMP revealed significant differences among the experimental groups [*F*(3,42) = 8.46, *P* = 0.0002]. *Post hoc* comparisons demonstrated a pronounced mitochondrial depolarization, reflected by an increased Δ*F*_535_/*F*_585_ ratio, in the EX527 group compared with control animals (*P* = 0.0317). Likewise, MetS animals exhibited a significant depolarization relative to the Con group (*P* = 0.002). Importantly, treatment with the sirtuin activator SRT1720 effectively normalized the MetS‐induced mitochondrial depolarization (*P* = 0.0013 vs. MetS), with no significant difference detected between the SRT1720 and Con groups (*P* > 0.9999).

ROS levels also differed significantly among groups [*F*(3,56) = 11.79, *P* < 0.0001]. *Post hoc* analyses revealed a marked increase in ROS production in Con + EX527 animals compared with controls (*P* < 0.0001). Likewise, MetS induced a significant elevation in ROS levels (*P* = 0.0003 vs. Con). In contrast, SRT1720 treatment effectively prevented the MetS‐associated increase in ROS production (*P* = 0.0217 vs. MetS), and ROS levels in the SRT1720 group were comparable to those observed in control animals (*P* = 0.7677).

### 3.3 Metabolic stress disrupts intracellular Ca^2+^ homeostasis

**FIGURE 3:**
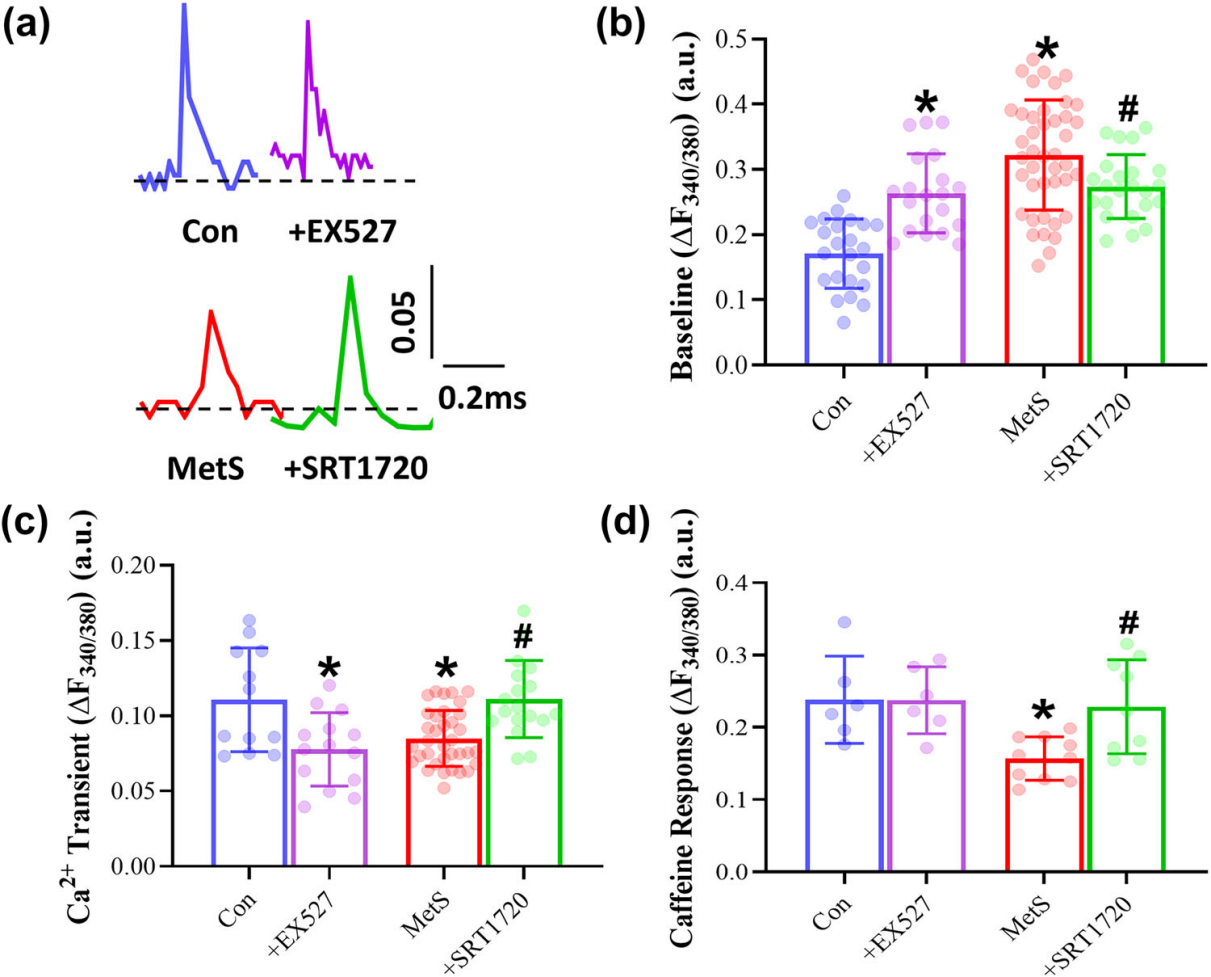
Intracellular Ca^2+^ homeostasis in isolated cardiomyocytes from experimental groups. (a) Representative [Ca^2+^]_i_ recording from cardiomyocytes. (b) Baseline [Ca^2+^]_i_. (c) Electrically evoked Ca^2+^ transients during cardiomyocyte contraction. (d) Peak [Ca^2+^]_i_ response following caffeine application, reflecting total sarcoplasmic reticulum Ca^2+^ content. Data are presented as the mean ± SD. Individual data points represent individual cells obtained from independent cell preparations. Sample sizes were as follows: baseline [Ca^2+^]_i_ and electrically evoked Ca^2+^ transients, Con (*n* = 12 cells), EX527 (*n* = 14 cells), MetS (*n* = 35 cells) and SRT1720 (*n* = 20 cells); caffeine response experiments, Con (*n* = 6 cells), EX527 (*n* = 7 cells), MetS (*n* = 10 cells) and SRT1720 (*n* = 9 cells). *P < 0.05 vs. Con; #*P* < 0.05 vs. MetS. Abbreviations: Con, control; MetS, metabolic syndrome.

Baseline [Ca^2+^]_i_, electrically evoked Ca^2+^ transients and total sarcoplasmic reticulum (SR) Ca^2+^ content assessed by caffeine application are summarized in Figure 3.

Baseline [Ca^2+^]_i_ differed significantly among the experimental groups [*F*(3,104) = 24.74, *P* < 0.0001]. Both MetS and EX527 administration markedly increased resting cytosolic Ca^2+^ levels compared with controls (*P* < 0.0001 and *P* = 0.0001, respectively). Treatment with SRT1720 effectively normalized this elevation (*P* = 0.0348 vs. MetS).

Ca^2+^ transient amplitudes also differed significantly across groups [*F*(3,75) = 8.72, *P* < 0.0001]. MetS animals exhibited a reduced Ca^2+^ transient amplitude relative to control cardiomyocytes (*P* = 0.0109), and EX527 treatment further depressed transient amplitude compared with controls (*P* = 0.0046). Notably, Ca^2+^ transient amplitude was significantly enhanced in MetS animals treated with SRT1720 compared with the MetS group (*P* = 0.0019), indicating a partial restoration of Ca^2+^ cycling.

Caffeine‐evoked Ca^2+^ release, reflecting total SR Ca^2+^ content, also differed among groups [*F*(3,27) = 5.17, *P* = 0.0060]. No significant difference was observed between Con and Con+EX527 groups (*P* > 0.9999). In contrast, MetS markedly blunted the caffeine‐induced Ca^2+^ response compared with controls (*P* = 0.0230). Importantly, SRT1720 treatment restored SR Ca^2+^ release in MetS animals (*P* = 0.0249 vs. MetS).

### 3.4 Metabolic stress alters the expression and phosphorylation of Ca^2+^‐handling proteins

**FIGURE 4:**
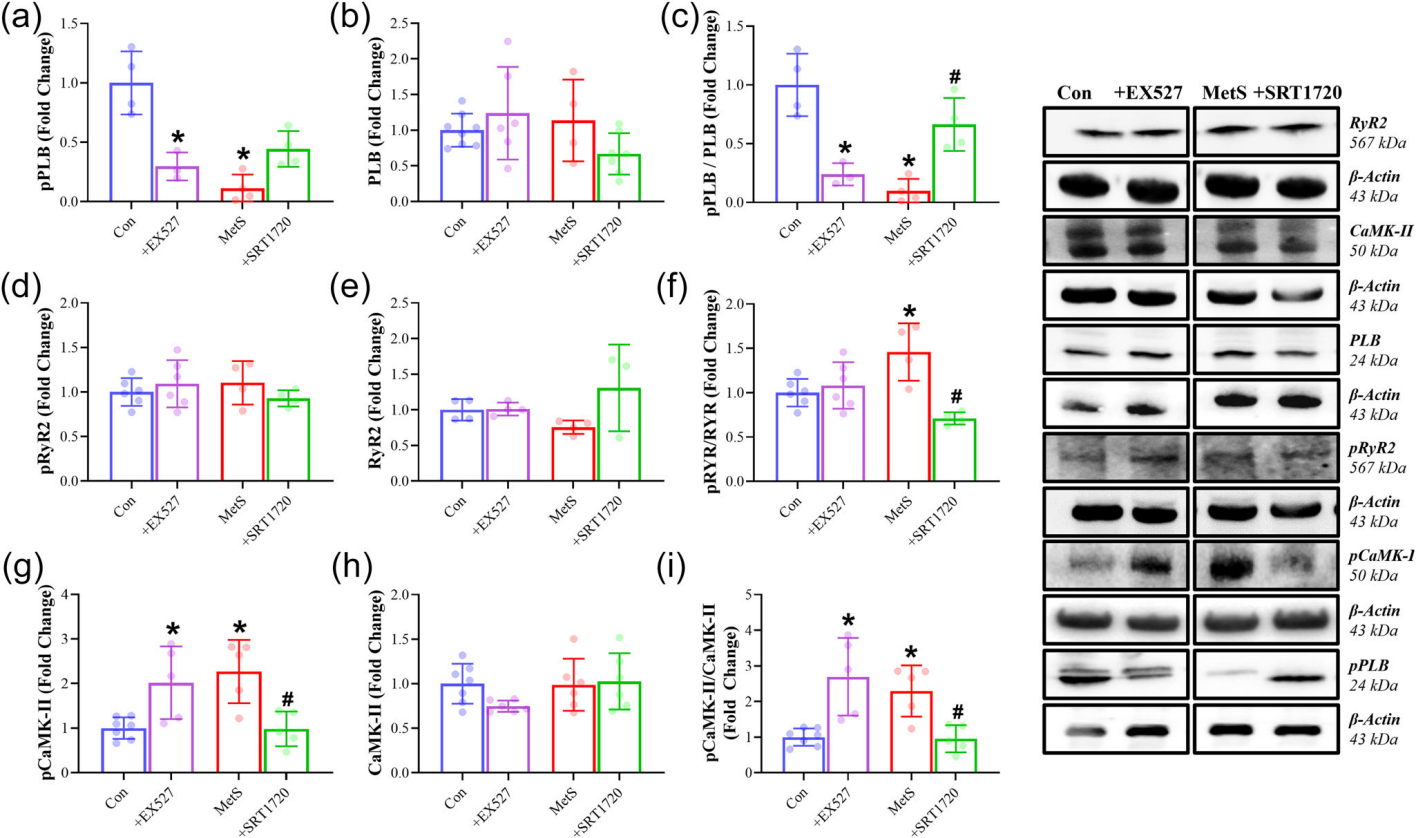
Alterations in Ca^2+^‐handling proteins during metabolic stress. Representative western blot images and quantitative analyses of PLB phosphorylation and expression, SERCA2, RyR phosphorylation and expression, and CaMKII phosphorylation and expression in isolated cardiomyocytes from experimental groups. (a) Phosphorylated phospholamban (p‐PLB; Ser16). (b) Total phospholamban (PLB). (c) p‐PLB/PLB ratio. (d) Phosphorylated ryanodine receptor (p‐RyR; Ser2808). (e) Total ryanodine receptor (RyR). (f) p‐RyR/RyR ratio. (g) Phosphorylated CaMKII (p‐CaMKII; Thr286). (h) Total CaMKII. (i) p‐CaMKII/CaMKII ratio. Data are presented as the mean ± SD. Individual data points represent independent biological samples. Sample sizes were as follows: p‐PLB, Con (*n* = 4), EX527 (*n* = 3), MetS (*n* = 4) and SRT1720 (*n* = 4); PLB, Con (*n* = 8), EX527 (*n* = 6), MetS (*n* = 4) and SRT1720 (*n* = 7); p‐RyR, Con (*n* = 6), EX527 (*n* = 6), MetS (*n* = 4) and SRT1720 (*n* = 4); RyR, Con (*n* = 4), EX527 (*n* = 4), MetS (*n* = 4) and SRT1720 (*n* = 3); p‐CaMKII, Con (*n* = 7), EX527 (*n* = 5), MetS (*n* = 5) and SRT1720 (*n* = 5); CaMKII, Con (*n* = 7), EX527 (*n* = 6), MetS (*n* = 6) and SRT1720 (*n* = 6). *P < 0.05 vs. Con; #*P* < 0.05 vs. MetS. Abbreviations: CAMKII, Ca^2+^/calmodulin‐dependent protein kinase II; Con, control; MetS, metabolic syndrome; PLB, phospholamban; RyR, ryanodine receptor; SERCA2, sarcoplasmic reticulum Ca^2+^‐ATPase 2.

Proteins involved in cardiomyocyte Ca^2+^ handling, including SERCA2, ryanodine receptor (RyR), phospholamban (PLB), CaMKII and their phosphorylated forms, were analysed by western blotting. Corresponding phosphorylation ratios are presented in Figure 4.

Total PLB protein levels did not differ significantly among experimental groups [Kruskal–Wallis test, *H*(3) = 5.494, *P* = 0.139]. In contrast, phosphorylated PLB (p‐PLB) levels showed significant differences across groups [Kruskal–Wallis test, *H*(3) = 11.85, *P* = 0.0001]. Both MetS and EX527 treatments markedly reduced p‐PLB levels compared with control cells (*P* = 0.009 and *P* = 0.0281, respectively), and p‐PLB levels were not restored by SRT1720 treatment (*P* = 0.0578 vs. MetS). Analysis of the phosphorylation ratio revealed significant differences among groups [Kruskal–Wallis test, *H*(3) = 11.87, *P* = 0.0001]. The p‐PLB/PLB ratio was significantly reduced in the EX527 and MetS groups compared with controls (*P* = 0.0248 and *P* = 0.0016, respectively), whereas SRT1720 treatment significantly increased PLB phosphorylation relative to MetS animals (*P* = 0.0269).

SERCA2 protein expression did not differ among the experimental groups [Kruskal–Wallis test, *H*(3) = 3.133, *P* = 0.3716; data not shown].

Total RyR and phosphorylated RyR (p‐RyR) protein levels were also comparable across groups [Kruskal–Wallis test, *H*(3) = 5.575, *P* = 0.1309 and *H*(3) = 1.905, *P* = 0.5924, respectively]. However, the p‐RyR/RyR phosphorylation ratio differed significantly among groups [Kruskal–Wallis test, *H*(3) = 11.38, *P* = 0.0098]. MetS animals exhibited a significant increase in RyR phosphorylation compared with controls (*P* = 0.0263), which was effectively reversed by SRT1720 treatment (*P* = 0.0011 vs. MetS).

Total CaMKII protein expression remained unchanged across groups [Kruskal–Wallis test, *H*(3) = 5.075, *P* = 0.1664]. In contrast, phosphorylated CaMKII (p‐CaMKII) levels differed significantly [Kruskal–Wallis test, *H*(3) = 10.18, *P* = 0.0171], with both EX527 and MetS treatments inducing a marked increase compared with controls (*P* = 0.0261 and *P* = 0.0095, respectively). SRT1720 treatment significantly reduced p‐CaMKII levels relative to MetS animals (*P* = 0.0284). Consistently, the p‐CaMKII/CaMKII ratio differed among groups [Kruskal–Wallis test, *H*(3) = 14.04, *P* = 0.0028], showing elevated phosphorylation in the EX527 and MetS groups (*P* = 0.012 and *P* = 0.0163 vs. Con) and normalization towards control levels following SRT1720 treatment (*P* = 0.0220 vs. MetS).

### 3.5 Metabolic stress activates apoptotic signalling pathways

**FIGURE 5:**
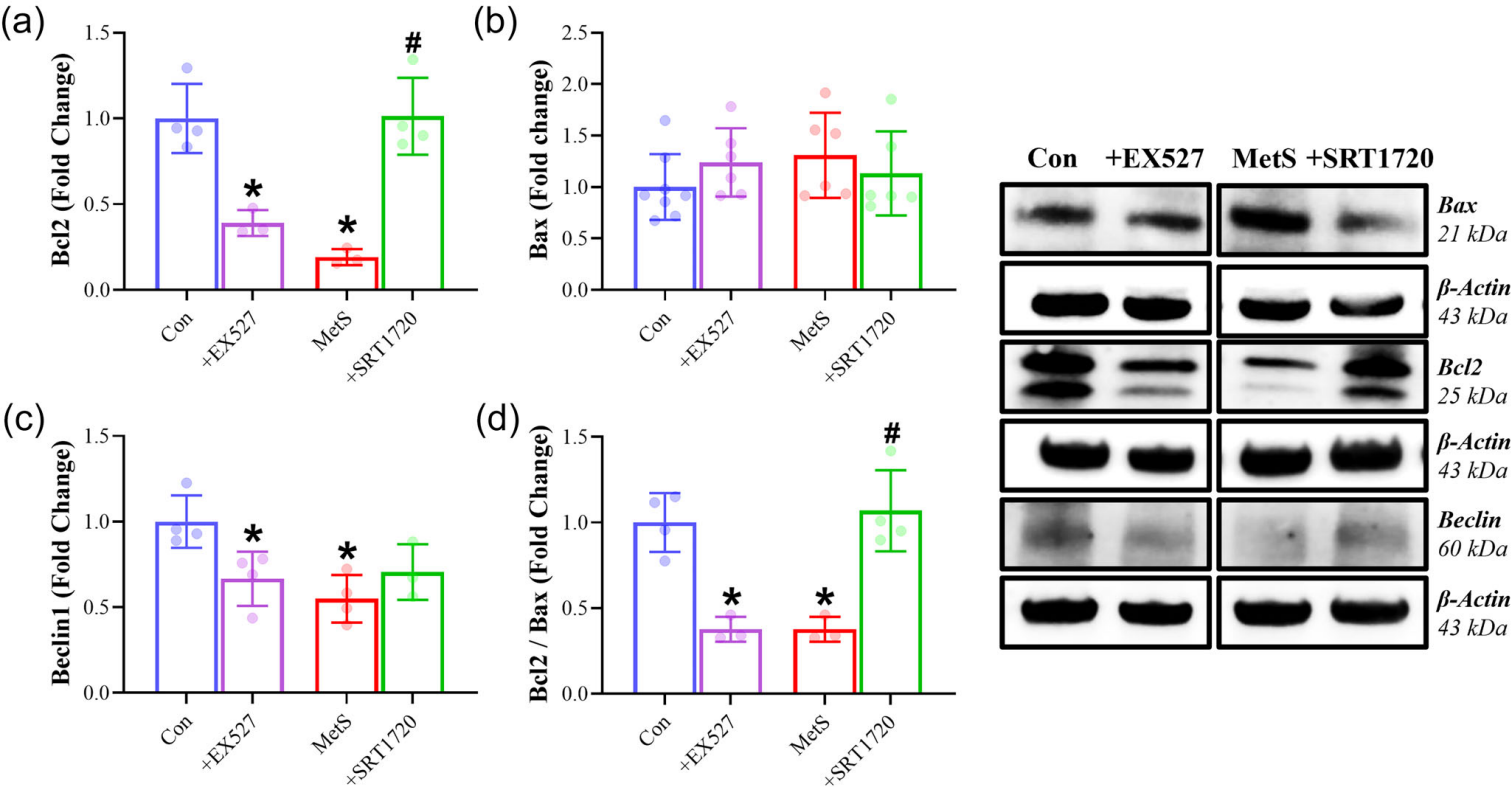
Activation of apoptotic signalling pathways in response to metabolic stress. Representative western blot images and quantitative analyses of anti‐apoptotic Bcl‐2, pro‐apoptotic Bax, and the apoptotic index expressed as the Bcl‐2/Bax ratio in isolated cardiomyocytes from experimental groups. (a) Bcl‐2. (b) Bax. (c) Bcl‐2/Bax ratio. (d) Beclin‐1 protein expression as an indicator of autophagy‐related signalling. Data are presented as the mean ± SD. Individual data points represent independent biological samples. Sample sizes were as follows: Bcl‐2, Con (*n* = 4), EX527 (*n* = 3), MetS (*n* = 3) and SRT1720 (*n* = 4); Bax, Con (*n* = 7), EX527 (*n* = 6), MetS (*n* = 6) and SRT1720 (*n* = 6); Beclin‐1, Con (*n* = 4), EX527 (*n* = 4), MetS (*n* = 4) and SRT1720 (*n* = 3). *P < 0.05 vs. Con; #*P* < 0.05 vs. MetS. Abbreviations: Con, control; MetS, metabolic syndrome.

Proteins involved in pro‐ and anti‐apoptotic signalling pathways, and the apoptotic index, expressed as the Bcl‐2/Bax ratio, are presented in Figure 5.

Anti‐apoptotic Bcl‐2 protein expression differed significantly among experimental groups [Kruskal–Wallis test, *H*(3) = 10.40, *P* = 0.0012], whereas pro‐apoptotic Bax levels remained comparable across groups [Kruskal–Wallis test, *H*(3) = 4.37, *P* = 0.2242]. Bcl‐2 expression was markedly reduced in both MetS and EX527 groups compared with control animals (*P* = 0.0098 and *P* = 0.0039, respectively). In contrast, SRT1720 treatment significantly increased Bcl‐2 levels relative to the MetS group (*P* = 0.0062).

Consistent with these findings, the Bcl‐2/Bax ratio differed significantly among groups [Kruskal–Wallis test, *H*(3) = 9.664, *P* = 0.0042]. This ratio was substantially decreased in both MetS and EX527 groups compared with controls (*P* = 0.0279 for both), indicating enhanced apoptotic susceptibility. Importantly, SRT1720 treatment markedly increased the Bcl‐2/Bax ratio relative to MetS animals (*P* = 0.0279), suggesting attenuation of apoptotic signalling.

Expression of Beclin‐1, a key regulator of autophagy, also differed significantly among experimental groups [Kruskal–Wallis test, *H*(3) = 9.213, *P* = 0.0086]. Beclin‐1 levels were significantly reduced in both EX527‐ and MetS‐treated animals compared with controls (*P* = 0.03298 and *P* = 0.0034, respectively). However, SRT1720 treatment did not significantly alter Beclin‐1 expression, with levels remaining comparable between the MetS and SRT1720 groups (*P* = 0.4208).

## 4 DISCUSSION

In the present study, we demonstrate that high sucrose‐induced MetS is associated with pronounced disturbances in cardiomyocyte Ca^2+^ handling, accompanied by increased oxidative stress, mitochondrial dysfunction and enhanced apoptotic signalling. Importantly, modulation of sirtuin activity differentially influenced these pathways. Pharmacological inhibition of SIRT1 with EX527 exacerbated abnormalities in Ca^2+^ homeostasis, mitochondrial function and apoptotic signalling in control cardiomyocytes, whereas SIRT1 activation with SRT1720 partly restored Ca^2+^ handling, redox balance and mitochondrial function while attenuating apoptosis in MetS cardiomyocytes. Although our findings are based on pharmacological modulation of sirtuin signalling, they collectively support a role for sirtuins in coordinating the crosstalk between metabolic stress, mitochondrial dysfunction and Ca^2+^ homeostasis in the heart.

To establish a MetS phenotype, BALB/c mice were exposed to a 32% sucrose solution *ad libitum* for 6 months. This dietary intervention resulted in significantly elevated fasting blood glucose levels, impaired glucose tolerance and increased serum triglyceride concentrations, consistent with the development of dyslipidaemia and systemic metabolic dysfunction. Notably, sucrose‐fed mice did not exhibit a significant increase in terminal body weight compared with control animals. Rodents are known to reduce chow consumption markedly when excess calories are provided via drinking water (Spector & Smith, 1984), a phenomenon that was also observed in the present study. Despite reduced solid food intake, the estimated total caloric intake of MetS animals remained significantly higher than that of control mice, indicating a sustained positive energy balance (data not shown). Although energy expenditure and body composition were not assessed directly, similar dissociations between body weight gain and metabolic impairment have been consistently reported in sucrose‐ or fructose‐fed rodent models, in which metabolic abnormalities and increased adiposity develop in the absence of excess weight gain (Jürgens et al., 2005; Kendig et al., 2015; Togo et al., 2019).

The primary determinants of intracellular Ca^2+^ homeostasis were assessed systematically. Basal [Ca^2+^]_i_ was significantly elevated in MetS cardiomyocytes compared with control cells, accompanied by a marked reduction in Ca^2+^ transient amplitude. This pattern is consistent with numerous studies reporting impaired excitation–contraction coupling and delayed Ca^2+^ clearance in cardiomyocytes from sucrose‐ or fructose‐fed rodent models of MetS and insulin resistance (Balderas‐Villalobos et al., 2013; Davidoff et al., 2004; Dutta et al., 2001; Fernández‐Miranda et al., 2019; Hintz & Ren, 2002; Okatan et al., 2016; Vasanji et al., 2006; Wold et al., 2005). In this context, altered RyR2 phosphorylation and CaMKII hyperactivation (often driven by oxidative stress) have been implicated as major contributors to SR Ca^2+^ leak and arrhythmogenic Ca^2+^ handling in prediabetic and MetS models (Belke et al., 2004; Pereira et al., 2006; Romero‐García et al., 2020; Sommese et al., 2016).

A similar pattern of elevated resting [Ca^2+^]_i_ and blunted Ca^2+^ transients was observed following pharmacological inhibition of SIRT1 with EX527 in control cardiomyocytes, supporting the hypothesis that reduced sirtuin activity contributes directly to Ca^2+^ dysregulation. This observation aligns with previous reports demonstrating that impaired SIRT1 signalling disrupts SERCA2a function predominantly through post‐translational mechanisms rather than alterations in protein expression (Balderas‐Villalobos et al., 2013; Sulaiman et al., 2010; Wold et al., 2005). In contrast, activation of SIRT1 signalling with SRT1720 in MetS cardiomyocytes normalized both basal [Ca^2+^]_i_ and Ca^2+^ transient amplitude, extending earlier findings that enhancement of sirtuin activity improves Ca^2+^ cycling and contractile performance in metabolically compromised myocardium (Sulaiman et al., 2010).

Assessment of total SR Ca^2+^ content using caffeine‐evoked Ca^2+^ release revealed a significant reduction in SR Ca^2+^ load in MetS cardiomyocytes. This finding is consistent with reports describing diminished SR Ca^2+^ content and increased diastolic Ca^2+^ leak in diabetic and metabolic cardiomyopathy models (Belke et al., 2004; Okatan et al., 2016; Pereira et al., 2006). However, other studies in sucrose‐fed MetS models have reported preserved SR Ca^2+^ content despite impaired Ca^2+^ cycling, suggesting that SR Ca^2+^ load is highly dependent on disease duration, dietary composition and the balance between SERCA‐mediated Ca^2+^ uptake and RyR‐mediated Ca^2+^ leak (Balderas‐Villalobos et al., 2013; Fernández‐Miranda et al., 2019; Wold et al., 2005). Importantly, although SR Ca^2+^ load was restored by SRT1720 treatment in MetS cardiomyocytes, acute SIRT1 inhibition with EX527 did not significantly alter the caffeine response in control cells. The differential effects of EX527 and MetS on SR Ca^2+^ content suggest that acetylation‐dependent regulation of calcium homeostasis might differ between physiological and pathological conditions. Several mechanisms might explain why acute SIRT1 inhibition increased resting cytosolic Ca^2+^ without significantly altering SR Ca^2+^ load, whereas MetS affected both parameters. Acute SIRT1 inhibition might increase sarcolemmal Ca^2+^ influx independently of SR function, thereby elevating basal cytosolic Ca^2+^ without substantially affecting SR Ca^2+^ stores (Bers, 2008; Tuncay et al., 2023). In addition, altered acetylation status might influence intracellular Ca^2+^ buffering systems, including cytosolic Ca^2+^‐binding proteins and mitochondrial Ca^2+^‐uptake pathways (Sommese et al., 2016). Furthermore, RyR2 phosphorylation was significantly increased in MetS cardiomyocytes but remained unchanged following EX527 treatment, suggesting that enhanced RyR2‐mediated SR Ca^2+^ leak might contribute to the reduction in SR Ca^2+^ content observed in MetS (Romero‐García et al., 2020; Sommese et al., 2016). Finally, the relatively short duration of EX527 exposure is unlikely to reproduce the chronic metabolic, transcriptional and epigenetic remodelling associated with long‐term MetS (Capasso et al., 2025; Kinchen et al., 2008; Schug & Li, 2011). Consequently, acute pharmacological inhibition of SIRT1 is likely to reproduce only a subset of the mechanisms contributing to Ca^2+^ dysregulation in MetS. The lack of effect of EX527 in control cardiomyocytes, together with the restorative effect of SRT1720 in MetS cells, suggests that acetylation‐dependent regulation of calcium handling might become more relevant in conditions of metabolic stress. This observation raises the possibility that the molecular targets and functional consequences of sirtuin‐mediated deacetylation differ between physiological and pathological states.To determine whether impaired Ca^2+^ homeostasis was associated with cellular injury and energetic dysfunction, we next examined oxidative stress, MMP and apoptotic signalling in cardiomyocytes. MetS cardiomyocytes exhibited a marked increase in ROS production accompanied by mitochondrial depolarization and pro‐apoptotic alterations, indicating that metabolic stress promotes cardiomyocyte injury through mitochondria‐dependent cell death pathways. These findings are consistent with previous reports implicating mitochondrial dysfunction and oxidative stress in the pathogenesis of metabolic cardiomyopathy (Balderas‐Villalobos et al., 2013; Sommese et al., 2016). Mitochondrial ROS have been shown to disrupt Ca^2+^ handling directly by inducing oxidative modifications of Ca^2+^‐handling proteins and activating redox‐sensitive kinases, such as CaMKII (Erickson et al., 2011; Sommese et al., 2016). Importantly, pharmacological inhibition of SIRT1 in control cardiomyocytes recapitulated several features of the MetS phenotype, whereas activation of sirtuin signalling with SRT1720 in MetS cardiomyocytes attenuated oxidative stress, partly restored MMP and reduced apoptotic signalling. Together, these observations underscore a central regulatory role for sirtuin signalling in protecting cardiomyocytes against metabolic stress‐induced mitochondrial dysfunction and cell injury.

Analysis of key Ca^2+^‐handling proteins revealed several mechanistically informative alterations in MetS cardiomyocytes. Notably, total SERCA2a protein expression was comparable among experimental groups, consistent with numerous previous reports indicating preserved SERCA2a abundance in metabolic and diabetic cardiomyopathy despite functional impairment (Balderas‐Villalobos et al., 2013; Okatan et al., 2016; Vasanji et al., 2006; Wold et al., 2005).

As the primary regulator of SERCA, total PLB protein levels were likewise unchanged; however, PLB phosphorylation was markedly reduced both in MetS cardiomyocytes and in control cardiomyocytes treated with the SIRT1 inhibitor EX527. Because PLB phosphorylation relieves its inhibitory constraint on SERCA2a, reduced PLB phosphorylation provides a plausible molecular mechanism for the impaired SR Ca^2+^ reuptake and delayed cytosolic Ca^2+^ clearance observed during metabolic stress and sirtuin inhibition. The parallel effects of chronic metabolic stress and acute SIRT1 inhibition on PLB phosphorylation further support a regulatory role for sirtuin signalling in maintaining effective SERCA2a function.

Total RyR2 protein expression also remained unchanged across groups, whereas RyR2 phosphorylation was significantly increased in MetS cardiomyocytes and fully normalized by sirtuin activation with SRT1720. Enhanced RyR2 phosphorylation is known to increase diastolic SR Ca^2+^ leak, thereby contributing to elevated resting cytosolic Ca^2+^ levels and reduced total SR Ca^2+^ content. These findings are in agreement with previous studies implicating aberrant RyR2 phosphorylation, rather than altered channel expression, as a central driver of Ca^2+^ mishandling in metabolic and diabetic cardiomyopathy (Belke et al., 2004; Miranda et al., 2015; Okatan et al., 2016; Pereira et al., 2006; Romero‐García et al., 2020). The increased phosphorylation of ryanodine receptors without changing the RyR2 protein level observed in MetS cardiomyocytes compared with controls is consistent with enhanced SR Ca^2+^ leak during chronic metabolic stress. In contrast, acute SIRT1 inhibition with EX527 in control cardiomyocytes did not significantly alter RyR phosphorylation, indicating that basal RyR regulatory mechanisms are largely preserved in physiological conditions and might not be acutely dependent on SIRT1 activity. This finding suggests that elevated RyR phosphorylation in MetS reflects long‐term maladaptive signalling driven by metabolic and oxidative stress rather than direct, short‐term effects of SIRT1 inhibition. Accordingly, acute EX527 treatment is sufficient to impair Ca^2+^ cycling dynamics without inducing the sustained RyR hyperphosphorylation characteristic of the MetS phenotype (Belke et al., 2004; Pereira et al., 2006; Sommese et al., 2016).

CaMKII is a critical upstream regulator of multiple Ca^2+^‐handling proteins. Although total CaMKII expression was unchanged, CaMKII phosphorylation (hence kinase activity) was markedly increased in both MetS cardiomyocytes and EX527‐treated control cells and significantly reduced following SRT1720 treatment. Increased CaMKII activity provides a mechanistic explanation for the observed RyR2 hyperphosphorylation. The observed alterations in CaMKII phosphorylation might also provide a mechanistic link between acetylation‐dependent signalling and phosphorylation‐based regulation of Ca^2+^‐handling proteins. Recent evidence suggests that reduced SIRT1 activity can promote mitochondrial dysfunction and oxidative stress, which, in turn, enhances CaMKII oxidation and hyperphosphorylation, leading to downstream RyR2 phosphorylation and abnormal SR Ca^2+^ handling (Qiu et al., 2024). In this context, the increased CaMKII phosphorylation observed in both MetS and EX527‐treated cardiomyocytes might represent a downstream consequence of altered sirtuin‐dependent acetylation signalling rather than an isolated kinase abnormality. Furthermore, emerging evidence indicates bidirectional crosstalk between Ca^2+^‐dependent kinase signalling and SIRT1 regulation. CaMKKβ has been shown to phosphorylate SIRT1, thereby increasing its stability and catalytic activity, suggesting the existence of a reciprocal regulatory loop linking intracellular Ca^2+^ signalling and sirtuin activity (Wen et al., 2013). Together, these observations support the concept that acetylation‐ and phosphorylation‐dependent signalling pathways interact closely during the development of metabolic cardiomyopathy.

Interestingly, despite increased CaMKII phosphorylation, PLB phosphorylation was reduced in both MetS and EX527‐treated cardiomyocytes. A similar dissociation has been reported previously, and several non‐mutually exclusive mechanisms might account for this apparent discrepancy. First, metabolic stress might alter the subcellular targeting of CaMKII, favouring its localization to RyR2‐rich junctional domains while limiting its access to PLB (Erickson et al., 2011; Sommese et al., 2016; Vasanji et al., 2006). Second, increased activity of serine/threonine phosphatases, such as PP1 or PP2A, in conditions of metabolic stress and oxidative imbalance might preferentially dephosphorylate PLB despite elevated kinase activity (Belke et al., 2004; Bidasee et al., 2003; Davidoff et al., 2004). Third, impaired β‐adrenergic/PKA signalling (known to contribute substantially to PLB Ser16 phosphorylation) might further suppress PLB phosphorylation independently of CaMKII activation (Balderas‐Villalobos et al., 2013; Sulaiman et al., 2010; Sulaiman et al., 2010). Unfortunately, the present study was not designed to interrogate all components of this signalling cascade, and therefore cannot discriminate definitively among these mechanisms.

## 5 CONCLUSION

**FIGURE 6:**
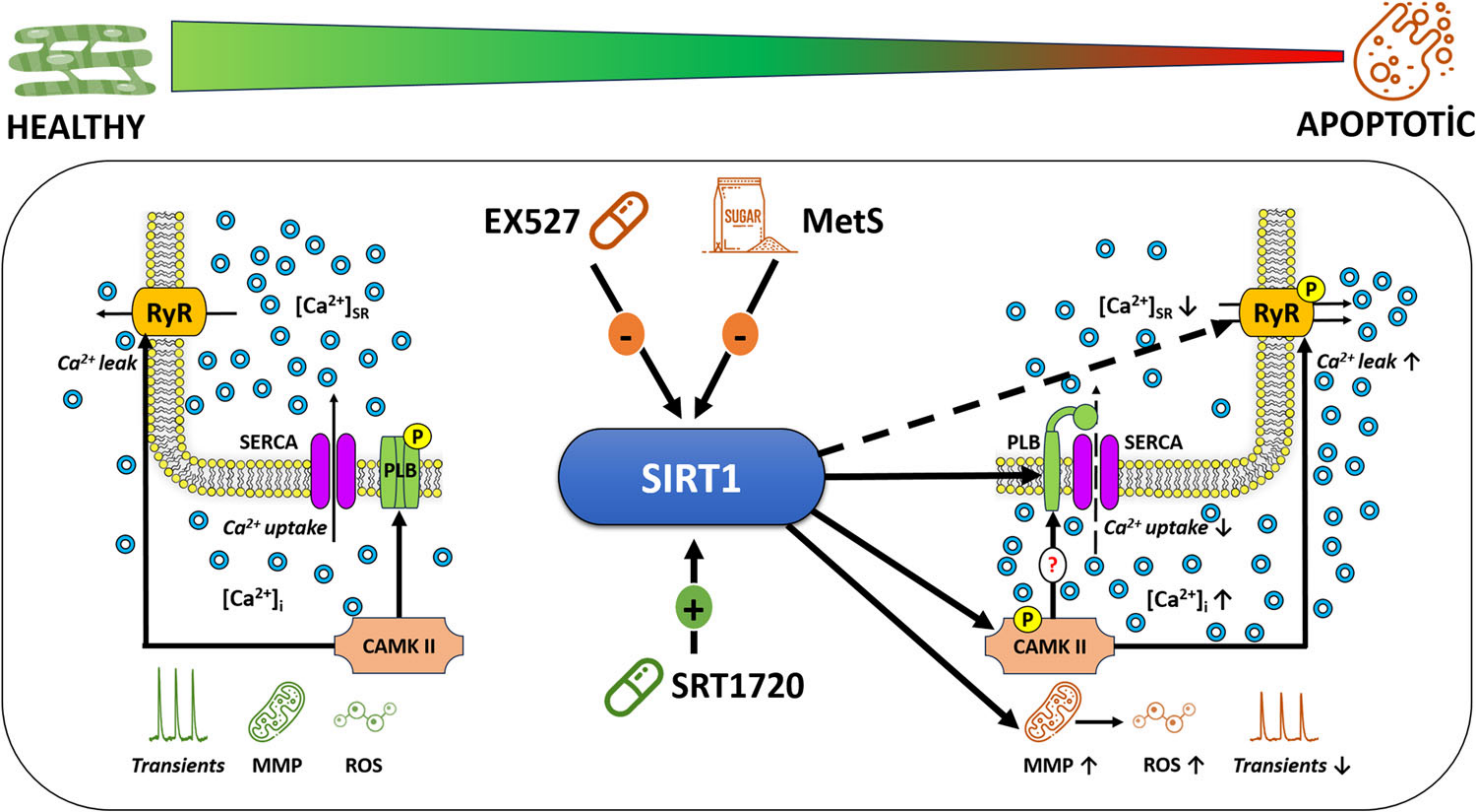
Schematic summary of the proposed mechanisms linking metabolic stress, sirtuin signalling and cardiomyocyte injury. The transition from a healthy cardiomyocyte to an apoptotic state is illustrated. Both metabolic stress and pharmacological inhibition of SIRT1 by EX527 suppress sirtuin activity, whereas SRT1720 activates this pathway. Reduced SIRT1 activity promotes CaMKII phosphorylation, a key regulator of intracellular Ca^2+^ homeostasis. Activated CaMKII enhances RyR2 phosphorylation, increasing SR Ca^2+^ leak and elevating cytosolic Ca^2+^ levels. Although CaMKII activation would normally increase PLB phosphorylation, PLB phosphorylation was markedly reduced in MetS and EX527‐treated cardiomyocytes, impairing SERCA‐mediated Ca^2+^ reuptake into the SR. This imbalance reduces SR Ca^2+^ content while increasing cytosolic Ca^2+^ concentration. The dissociation between CaMKII activation and PLB hypophosphorylation might reflect altered phosphatase activity during metabolic stress. In parallel, reduced SIRT1 activity impairs mitochondrial homeostasis, promoting mitochondrial dysfunction and increased ROS production, which further exacerbates Ca^2+^ dysregulation and apoptotic signalling. Abbreviations: CAMKII, Ca^2+^/calmodulin‐dependent protein kinase II; Con, control; MetS, metabolic syndrome; PLB, phospholamban; RyR, ryanodine receptor; ROS, reactive oxygen species; SERCA2, sarcoplasmic reticulum Ca^2+^‐ATPase 2; SR, sarcoplasmic reticulum.

In conclusion, the present study demonstrates that chronic sucrose‐induced metabolic stress profoundly disrupts cardiomyocyte Ca^2+^ homeostasis, mitochondrial function and redox balance, culminating in maladaptive Ca^2+^ handling and enhanced apoptotic signalling. Notably, these alterations occur despite preserved expression of key Ca^2+^‐handling proteins and are instead driven by dysregulated post‐translational modifications. Importantly, pharmacological modulation of sirtuin activity revealed a pivotal regulatory role for sirtuin signalling in these processes, highlighting sirtuin pathways as promising therapeutic targets in metabolic cardiomyopathy (Figure 6).

Although our findings provide strong evidence for a central role of post‐translational dysregulation in cardiomyocyte Ca^2+^ handling, the functional consequences were assessed primarily at the cellular level. Therefore, future studies using in vivo models are required to verify these results in physiological conditions. In addition, sirtuin signalling was modulated using pharmacological agents; although SRT1720 and EX527 are widely used, their potential off‐target effects cannot be excluded fully. Validation using alternative pharmacological modulators would further strengthen mechanistic specificity.

## AUTHOR CONTRIBUTIONS

Firat Akat: Performed the calcium measurements experiments, analysis, interpretation of the data, writing—original draft preparation. Leila Aryan: Performed the western blot experiments and analysed the data. Suatnur Şık: Performed the western blot experiments and analysed the data. Erkan Tuncay: Coordinated the study, methodology, investigation, analysis, interpretation of the data, writing—original draft preparation, writing—review and editing. All authors approved the final version of the manuscript and agree to be accountable for all aspects of the work in ensuring that questions related to the accuracy or integrity of any part of the work are appropriately investigated and resolved. All persons designated as authors qualify for authorship, and all those who qualify for authorship are listed.

## CONFLICT OF INTEREST

None declared.

## GENERATIVE AI STATEMENT

ChatGPT (OpenAI, GPT‐5.5) was used exclusively to improve the clarity, grammar and readability of the manuscript. All scientific content, study design, data analysis, interpretation of results, and conclusions were developed and verified by the authors. The authors take full responsibility for the content of the manuscript.

## Data Availability

The datasets generated and/or analysed during the study are available from the corresponding author upon reasonable request.
